# The acid adaptive tolerance response in *Campylobacter jejuni* induces a global response, as suggested by proteomics and microarrays

**DOI:** 10.1111/1751-7915.12302

**Published:** 2015-07-29

**Authors:** Athanasia Varsaki, Caroline Murphy, Alicja Barczynska, Kieran Jordan, Cyril Carroll

**Affiliations:** 1 Microbiology, School of Natural Sciences, National University of IrelandGalway, Ireland; 2 Teagasc Food Research Centre, MooreparkFermoy, Co. Cork, Ireland

## Abstract

*Campylobacter jejuni* CI 120 is a natural isolate obtained during poultry processing and has the ability to induce an acid tolerance response (ATR) to acid + aerobic conditions in early stationary phase. Other strains tested they did not induce an ATR or they induced it in exponential phase. *Campylobacter* spp. do not contain the genes that encode the global stationary phase stress response mechanism. Therefore, the aim of this study was to identify genes that are involved in the *C. jejuni* CI 120 early stationary phase ATR, as it seems to be expressing a novel mechanism of stress tolerance. Two-dimensional gel electrophoresis was used to examine the expression profile of cytosolic proteins during the *C. jejuni* CI 120 adaptation to acid + aerobic stress and microarrays to determine the genes that participate in the ATR. The results indicate induction of a global response that activated a number of stress responses, including several genes encoding surface components and genes involved with iron uptake. The findings of this study provide new insights into stress tolerance of *C. jejuni*, contribute to a better knowledge of the physiology of this bacterium and highlight the diversity among different strains.

## Introduction

*Campylobacter* spp. are Gram-negative, microaerophilic, spiral-shaped, motile bacteria. Their common habitat is the intestine of a wide variety of animals and avian species and they are pathogenic to humans. Globally, they are the most common bacterial cause of food-borne gastroenteritis. It has been estimated that as little as 500 cells of *Campylobacter jejuni* can initiate human disease (Black *et al*., 1988), which starts by colonization of the intestinal epithelium by the bacteria and then causes from a mild, watery to a severe, bloody diarrhoea (Skirrow and Blaser, 2000). *Campylobacter jejuni* infection can also be associated with the development of serious immune-mediated neurological sequelae, known as Guillain-Barré syndrome (reviewed in Janssen *et al*., 2008). Epidemiological studies have implicated transmission to humans by food-borne or water-borne routes, such as raw and undercooked poultry (Altekruse *et al*., 1999), raw milk (Evans *et al*., 1996) and tap water (Karagiannis *et al*., 2010).

*Campylobacter* spp. are thought to be fragile organisms outside of the host and lack many of the recognized stress response mechanisms found in other food-borne bacteria such as *Escherichia* and *Salmonella* (Ramos *et al*., 2001; Park, 2002). Even though there is a general lack of understanding of the physiology of *Campylobacter* spp. with regards to their ability to survive environmental stress, various survival mechanisms have been suggested for them. These mechanisms include the entry into a viable but non-cultivable state (Rollins and Colwell, 1986), the transition from rod to coccoid shape (Moran and Upton, 1987), the stationary phase survival mechanism (Martínez-Rodriguez *et al*., 2004), the extracellular signalling mechanism (Murphy *et al*., 2003a), the adaptive tolerance response (Murphy *et al*., 2003b), the high degree of genetic heterogeneity (Wassenaar and Newell, 2000) and the biofilm formation (Joshua *et al*., 2006).

One important stress condition encountered by gastrointestinal pathogens is surviving the acid pH environment in the stomach upon ingestion and within the phagosomes and phagolysosomes of the intestinal epithelial cells. Adaptation and survival at low pH can be important towards the development of disease by the gastrointestinal pathogens. Many bacteria, like *Salmonella enterica* (Tiwari *et al*., 2004)*, Streptococcus sobrinus* (Nascimento *et al*., 2004)*, Listeria monocytogenes* (Davis *et al*., 1996) and *Lactobacillus casei* (Zhang *et al*., 2012), have developed a mechanism known as adaptive tolerance response (ATR), during which a sub-lethal stress induces an adaptive response and provides protection towards subsequent exposure to a lethal stress (Goodson and Rowbury, 1989). The only reference available involving an ATR in *C. jejuni* is the case of *C. jejuni* CI 120; a natural isolate obtained during poultry processing, which has an increased acid resistance when compared with *C. jejuni* culture collection strain NCTC 11168 (Murphy *et al*., 2003b). *C. jejuni* CI 120 has survived the aerobic and heat stress imposed during processing, probably by inducing survival mechanisms, as it also has the ability to use extracellular signalling mechanisms in order to induce tolerance to stress factors (Murphy *et al*., 2003a).

In the current study, further characterization of the ATR observed in *C. jejuni* CI 120 was undertaken using a combination of transcriptomic and proteomic analyses. In particular, two-dimensional (2D) electrophoresis, in conjunction with peptide mass fingerprinting and microarrays, was used to identify genes that participate in the *C. jejuni* CI 120 acid stress response and in that way broaden the understanding of the survival of *C. jejuni* under hostile environmental conditions.

## Results

### *Campylobacter jejuni* CI 120 induces an ATR in early stationary phase under acid + aerobic stress

**Figure 1:**
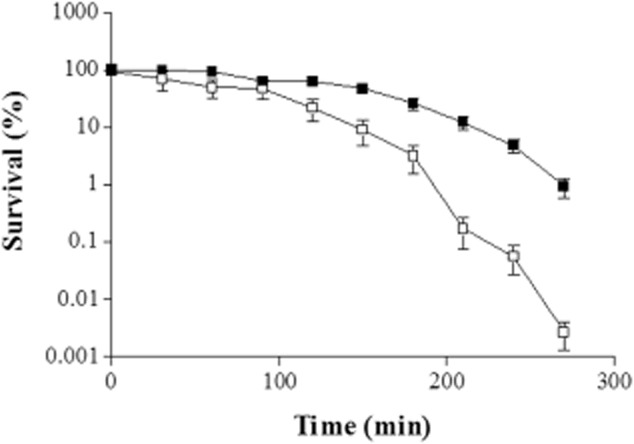
Typical survival curve of challenged *C. jejuni* CI 120 in early stationary phase. Open squares: unadapted cells, solid squares: challenged cells. Average values are shown with the standard deviation as error bars (*n* = 6).

**Figure 2:**
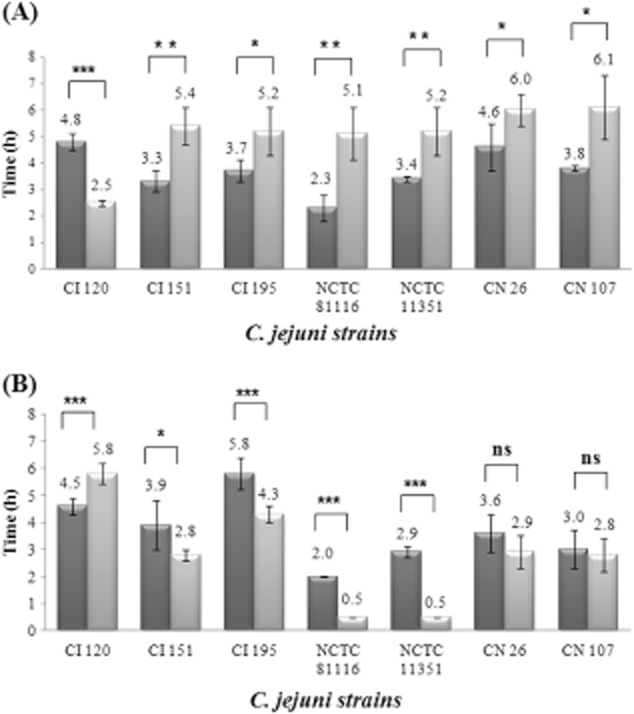
Induction of an adaptive tolerance response in different *C. jejuni* strains. Average values of the time (h) taken for the survivors to reach 0.1% of the initial population [t_S(0.1%)_] are shown with standard deviation as error bars (*n* = 6).A. Results of challenged cells in mid-exponential phase.B. Results of challenged cells in early stationary phase. Dark gray columns: unadapted cells, light gray columns: challenged cells.***Extremely significant difference (*P* < 0.001).**Very significant difference (*P* < 0.01).*Significant difference (*P* < 0.05).ns: Not significant difference (*P* > 0.05).

Seven (7) *C. jejuni* isolates were examined with respect to their ability to induce an ATR to acid + aerobic conditions in either mid-exponential or early stationary phase. Survival curves were generated from challenged (cultures whose pH was first dropped to pH 5.5 for 5 h under aerobic conditions and then challenged at pH 4.5) and unadapted cells (cultures whose pH was dropped directly to 4.5) of each *C. jejuni* strain tested, in mid-exponential and early stationary phase. A typical survival curve, where the results expressed are the average ± standard deviation (*n* = 6), is shown in Fig. 1. The Weibull survival model was used to statistically analyse the survival curves (Irwin *et al*., 1994). The time (h) taken for the survivors to reach 0.1% of the initial population [t_S(0.1%)_] was calculated from the survival curves of each *C. jejuni* strain tested, and the results (Fig. 2A and B) are expressed as the average ± standard deviation (*n* = 6). Data were analysed with a two-tailed student *t*-test and the significance of the differences are shown with asterisks in Fig. 2A and B. When cells were treated in mid-exponential phase (Fig. 2A), the t_S(0.1%)_ was longer for challenged cells, indicating that the majority of isolates induced an ATR in mid-exponential phase. On the other hand, in early stationary phase (Fig. 2B), the t_S(0.1%)_ was equal or longer for unadapted cells, indicating that isolates did not induce an ATR in early stationary phase. The exception to this was strain CI 120. For this strain in mid-exponential phase, the unadapted cells had t_S(0.1%)_ = 4.8 h instead of 2.5 h for the challenged cells, and in early stationary phase, the challenged cells had t_S(0.1%)_ = 5.8 h instead of 4.5 h for the unadapted cells. These results indicate that *C. jejuni* CI 120 was the only one of the strains tested that induced an ATR in early stationary and not in mid-exponential phase.

### The induction of an ATR in *C. jejuni* CI 120 alters the protein expression profile

**Figure 3:**
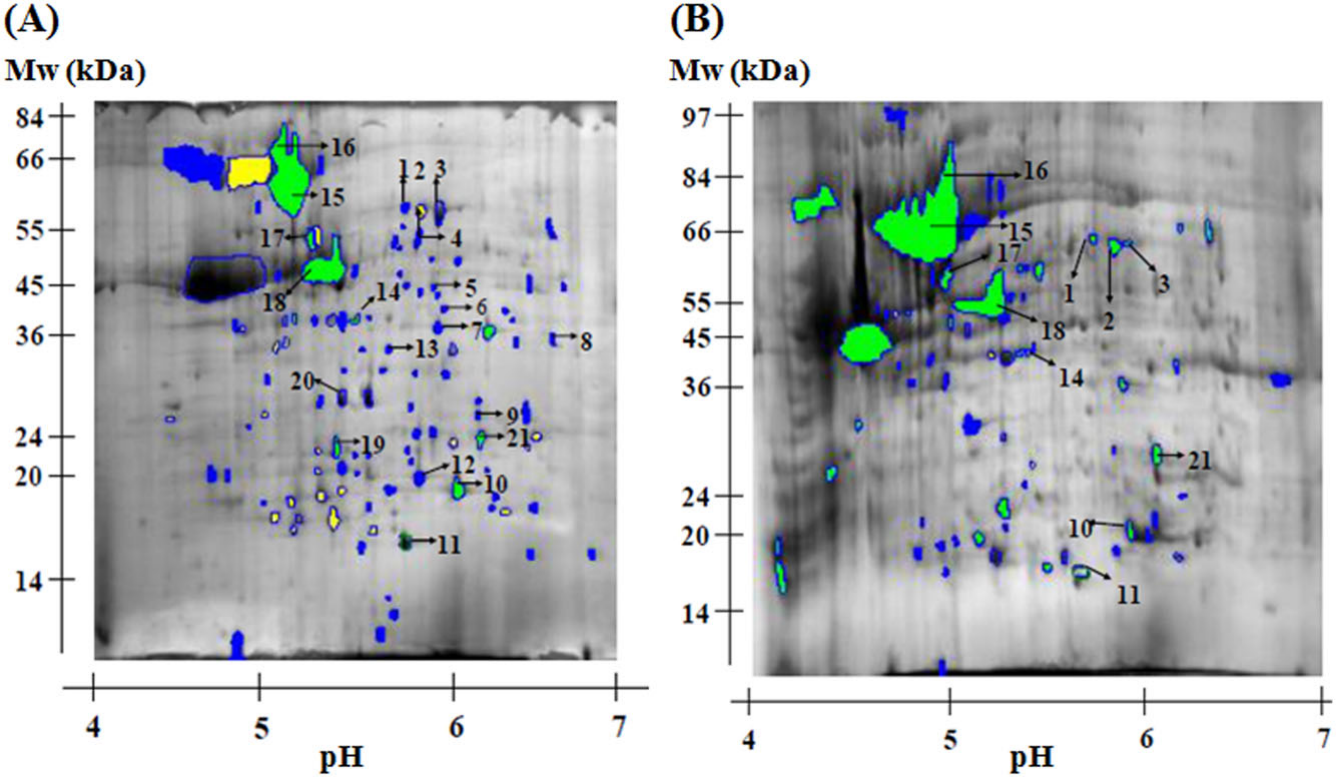
A. Image analysis of an average of triplicates *C. jejuni* CI 120 proteins from early stationary phase adapted cells which were compared with triplicates *C. jejuni* CI 120 proteins from early stationary phase control cells, normalized using spot number 11 (Chew protein). Proteins 1–21 were identified using MALDI-TOF mass spectrometry (see Table 1). B. Image analysis of an average of triplicates *C. jejuni* CN 107 proteins from early stationary phase adapted cells which were compared with triplicates *C. jejuni* CN 107 proteins from early stationary phase control cells and normalized using spot number 11. Arrowed spots had similar molecular mass and pI to the same numbered spots in gel shown in 3A, identified using MALDI-TOF mass spectrometry (see Table 1). Colour coding: proteins detected only after ATR (blue), upregulated proteins (green), downregulated proteins (yellow) and unchanged proteins (blue open circle).

Two-dimensional gels were used to compare protein expression profiles between adapted (cultures that were grown at 42°C to early stationary phase and then their pH was dropped to pH 5.5 for 5 h) and control cells (cultures grown at 42°C to early stationary phase) of *C. jejuni*. Analysis of cytosolic proteins in the pH range 3–10 revealed few proteins at the basic end (data not shown). Adapted cells exhibited a protein profile with marked difference from that displayed by control cells in the pH range 4–7. Consequently, in all subsequent analyses, only gels in the pH range 4–7 were produced. *Campylobacter jejuni* strains CI 120 and CN 107 were used for the proteomic analysis, as the former induced an ATR in early stationary phase, whereas the latter was unable to induce an ATR under the conditions used (Fig. 2A and B). Figure 3 shows a reference map of proteins from *C. jejuni* CI 120 and CN 170 separated by 2D gel electrophoresis. In the case of CI 120 (Fig. 3A), 107 spots were identified; of these, 9 were upregulated (cut-off ≥ 1.5-fold change), 21 were downregulated (cut-off ≤ 0.7-fold change), 9 were unchanged, and 68 were detected only after the induction of an ATR to acid + aerobic conditions in early stationary phase. The latter spots may correspond to newly synthesized proteins or proteins present but undetected under the control conditions. Mass spectrometry was used to identify 21 proteins; 8 upregulated, 1 downregulated, 3 unchanged and 9 detected after induction of the ATR. The results are shown in Table 1. In the case of *C. jejuni* CN 107 (Fig. 3B), normalization was performed using the protein spot that had similar molecular mass and pI to spot 11 on the image analysis of *C. jejuni* CI 120 (Fig. 3A). A total of 68 spots were identified, and of these 27 were upregulated, 3 were downregulated, 6 were unchanged and 32 were detected after the exposure period in pH 5.5. Mass spectrometry was used to identify 21 proteins that had similar pI and molecular mass to the 21 protein-spots of CI 120. The results are shown in Table 1.

**Table 1:** *Campylobacter jejuni* CI 120 and CN 107 proteins identified from 2-D gel electrophoresis by MALDI-TOF mass spectrometry and the differential expression from control to adapted cultures

Spot No.	Protein	Gene locus	Gene name	Function	CI 120^a^	CN 107^b^
1	Trigger factor P2	*cj0193c*	*tig*	Chaperones, chaperonins, heat shock	Only detected after ATR	Upregulated
2	Trigger factor P1	*cj0193c*	*tig*	Chaperones, chaperonins, heat shock	Downregulated	Upregulated
3	Trigger factor P3	*cj0193c*	*tig*	Chaperones, chaperonins, heat shock	Unchanged	Upregulated
4	Elongation factor Tu [EF-TU]	*cj0470*	*tuf*	Protein translation/modification	Only detected after ATR	Not-expressed
5	Succinyl-coA snythetase	*cj0533*	*sucC*	Biosynthesis of secondary metabolites	Only detected after ATR	Not-expressed
6	Pse synthetase	*cj1317*	*pseI*	Amino sugar and nucleotide sugar metabolism	Only detected after ATR	Not-expressed
7	Putative pyridine nucleotide-disulfide oxidoreductase	*cj0559*	null	Pyrimidine metabolism	Only detected after ATR	Not-expressed
8	Dihydrodipicolinate synthase	*cj0806*	*dapA*	Amino acid biosynthesis	Only detected after ATR	Not-expressed
9	Putative methyltransferase	*cj1419c*	null	Miscellaneous	Only detected after ATR	Not-expressed
10	Alkylhydroperoxide reductase	*cj0334*	*ahpC*	Oxidative stress resistance	Upregulated	Upregulated
11	CheW protein	*cj0283c*	*chew*	Chemotaxis and mobility	Equalization	Equalization
12	OOCR subunit of 2-oxoglutarate:acceptor oxidoreductase	*cj0538*	*oorC*	Metabolic pathways	Only detected after ATR	Not-expressed
13	Acetyl-coenzyme A carboxylase carboxyl transferase subunit alpha	*cj0443*	*accA*	Fatty acid biosynthesis	Only detected after ATR	Not-expressed
14	RecA protein	*cj1673c*	*recA*	Homologous recombination	Upregulated	Upregulated
15	GroEL	*cj1221*	*groEL*	Chaperones, chaperonins, heat shock	Unknown^c^	Unknown^c^
16	DnaK	*cj0759*	*dnaK*	Chaperones, chaperonins, heat shock	Unknown^c^	Unknown^c^
17	ATP synthase F1 sector beta subunit	*cj0107*	*atpD*	Energy metabolism	Upregulated	Upregulated
18	Elongation factor [EF-TU]	*cj0470*	*tuf*	Protein translation/modification	Upregulated	Upregulated
19	*Campylobacter* oxidative stress regulation (CosR)	*cj0355c*	*cosR*	Signal transduction	Upregulated	Not-expressed
20	Elongation factor P (EF-P)	*cj0551*	*efp*	Cell envelope	Unchanged	Undetected
21	Phospho-ribosyl-aminoimidazole succinocarboxamide synthetase	*cj0512*	*purC*	Purines, pyrimidines, nucleosides and nucleotides	Upregulated	Upregulated

a Difference in expression from control to adapted CI 120.

b Difference in expression from control to adapted CN 107.

c Could not be determined as the entire spot was treated as one for the purposes of quantification.

null: refers to genes that are putative and no name has been assigned to them.

### Identification of *C. jejuni* acid-inducible genes by microarray

**Figure 4:**
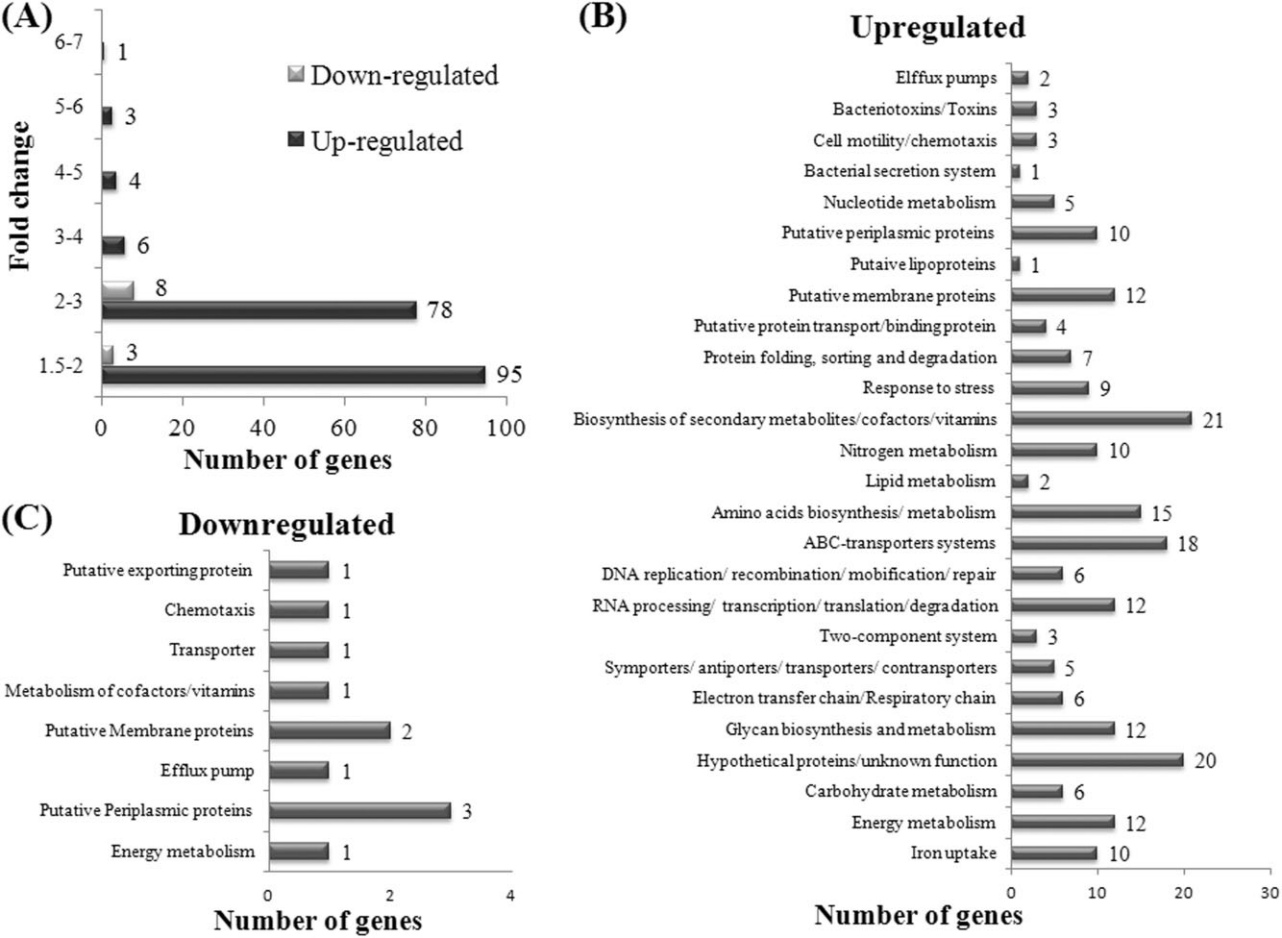
Summary of the effects of ATR adaptation on *C. jejuni* CI 120 gene expression.A. Distribution of genes whose expression was either increased or decreased in response to acid exposure, grouped according to fold changes in expression levels.B. Upregulated gene expression in *C. jejuni* CI 120 in response to ATR, classified into metabolic categories.C. Downregulated gene expression in *C. jejuni* CI 120 in response to ATR, classified into metabolic categories. Distribution was grouped according to Kyoto Encyclopedia of Genes and Genomes (KEGG). Some of the genes where classified in more than one metabolic category, as they participate in more than one metabolic route.

The differential gene expression of adapted and control *C. jejuni* CI 120 cells was performed using complementary ribonucleic acid (cRNA) microarrays, which was obtained from total RNA extracted from adapted and control cultures grown to early-stationary phase, as described in *Material and methods*. Each array contained 1594 genes and 4392 probes (up to three probes per gene). Under the experimental conditions, 1384 (86.8%) genes were expressed, of which 187 (13.5%) were upregulated, 11 (0.8%) were downregulated, and 1186 (85.7%) remained unchanged. A gene was considered to show altered expression when the *P*-value was ≤ 0.05. From the 187 upregulated genes, 51% were upregulated by 1.5–2-fold change and 42% by a 2–3-fold change (Fig. 4A). When classified into metabolic categories (Parkhill *et al*., 2000; Gundogdu *et al*., 2007), it became evident that genes encoding proteins involved in stress response and genes encoding putative membrane proteins and membrane transporters were considerably over-represented among the more strongly upregulated genes in adapted *C. jejuni* CI 120 cells (Fig. 4B). The downregulated genes were mainly genes encoding putative periplasmic, membrane or secretion proteins of unknown function (Fig. 4C). The most upregulated gene (six-fold change) was *cj1659* (*p19*), whose product is a membrane protein (19 kDa protein) potentially connected with iron uptake in *C. jejuni* and has also been suggested to be closely linked to oxidative stress defense (van Vliet *et al*., 2002). It is noteworthy that the majority of the strongly upregulated genes are related to iron regulation and transport. More specifically, the iron regulated ABC-transport system *cj0175c* (Tom-Yew *et al*., 2005) showed a three-fold change; the proposed ABC-transport system for the P19, *cj1660, cj1662, cj1663* (Holmes *et al*., 2005) was upregulated four-fold. These results show a direct relationship between the acid + aerobic stress and iron-regulated systems of *C. jejuni*. The complete list of the upregulated genes is given in Supporting information (Table S1). The complete list of the downregulated genes is given in Supporting information (Table S2).

### Validation of microarray expression data by quantitative real-time polymerase chain reaction

**Figure 5:**
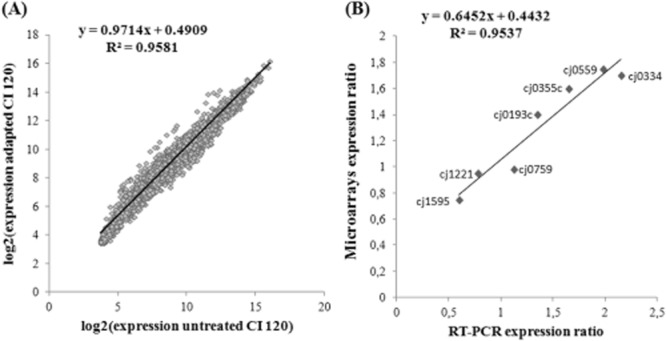
A. Scatter plot showing the correlation of gene expression between adapted and control *C. jejuni* CI 120 cells. Each point corresponds to the log_2_-transformed microarray expression of a single gene. B. Scatter plot showing the correlation between microarray and QRT-PCR expression ratios of seven genes comparing the expression between control and adapted cells. The best-fit linear regression line of each scatter plot is shown together with the R^2^ value and calculated equation for the slope.

The microarray data comparing the control cells with the adapted cells of *C. jejuni* CI 120 provide a direct measure of the transcriptome change. In order to assess the correlation between the two sets of measurements, the log_2_-transformed ratios of the control and adapted values for all genes on the microarray were taken and plotted against each other (Fig. 5A). There was a strong linear correlation between the gene expression changes. The strength of this correlation (R^2^ = 0.9581) indicates internal consistency in the dataset. The microarray data were also independently validated using quantitative real-time polymerase chain reaction (QRT-PCR) to measure the transcript levels of seven genes, randomly selected. Quantitative real-time PCR expression ratios were calculated with the ΔΔCt method (Livak and Schmittgen, 2001). The amplification efficiency (E) of each gene was derived as previously described (Rutledge and Côté, 2003), being within the range 1.9–2.1. The QRT-PCR experiments were independently repeated three times with distinct biological samples. The microarray and QRT-PCR expression were plotted against each other to assess the strength of the correlation between the measurements obtained by the two methods. The plot (Fig. 5B) shows a strong correlation between the measurements, with an R^2^ value of 0.953 indicating that the magnitude of expression changes detected by each method was highly similar.

### Comparison of 2D electrophoresis and microarray results

Since several proteins and/or genes were identified using proteomics and microarrays, respectively, the overlap among the proteins/genes identified by these two different approaches was examined. If these genes/proteins are truly involved in the ATR phenotype of this strain, one would expect an overlap in the proteins/genes identified by these two different approaches. In the 2D electrophoresis experiments, the proteins that appeared to be involved with the ATR were the ones that showed a difference in the protein expression profile from the control cells to the adapted cells in CI 120 and showed no difference in CN 107. The reason is that since CN 170 does not show any ATR in the conditions tested, only the proteins that are differentially expressed in CI 120 might be involved in the ATR observed. For a few number (3 out of 10) of the putative ATR-related proteins identified by 2D electrophoresis, the microarray data available had a *P*-value of > 0.05, and as a result the expression difference given from the microarrays is of no statistical value. In order to validate the accordance of the data from the 2D electrophoresis and the microarrays, QRT-PCR was performed and the expression ratios were calculated as described above for the validation of the microarrays. The results are shown in Table 2. In general, the two techniques (2D electrophoresis and microarrays) were in agreement.

**Table 2:** Comparison of the results in the differential expression from control to adapted cultures of *C. jejuni* CI 120 given by 2D-electrophoresis, microarrays and QRT-PCR

Spot No^a^.	Gene locus	Gene name	Function	2D-electrophoresis results^b^	Microarrays results^c^	QRT-PCR results^d^
5	*cj0533*	*sucC*	Biosynthesis of secondary metabolites	Only detected after ATR	R = 1.49, *P* = 0.37 (*P* value not acceptable)	R = 2.06 ± 0.09, *P* = 0.001 (Upregulated)
6	*cj1317*	*pseI*	Amino sugar and nucleotide sugar metabolism	Only detected after ATR	R = 1.05, *P* = 0.22 (*P* value not acceptable)	R = 2.31 ± 0.45, *P* = 0.01 (Upregulated)
7	*cj0559*	Null	Pyrimidine metabolism	Only detected after ATR	R = 1.75, *P* = 0.01 (Upregulated)	R = 1.98 ± 0.23, *P* = 0.01 (Upregulated)
8	*cj0806*	*dapA*	Amino acid biosynthesis	Only detected after ATR	R = 2.01, *P* = 0.02 (Upregulated)	R = 1.92 ± 0.18, *P* = 0.01 (Upregulated)
10	*cj0334*	*ahpC*	Oxidative stress resistance	Upregulated	R = 1.59, *P* = 0.004 (Upregulated)	R = 2.15 ± 0.47, *P* = 0.05 (Upregulated)
12	*cj0538*	*oorC*	Metabolic pathways	Only detected after ATR	R = 1.45, *P* = 0.17 (*P* value not acceptable)	R = 1.88 ± 0.24, *P* = 0.01 (Upregulated)
15	*cj1221*	*groEL*	Chaperones, chaperonins, heat shock	Unknown^e^	R = 0.95, *P* = 0.05 (Unchanged)	R = 0.78 ± 0.08, *P* = 0.01 (Unchanged)
16	*cj0759*	*dnaK*	Chaperones, chaperonins, heat shock	Unknown^e^	R = 0.95, *P* = 0.05 (Unchanged)	R = 1.12 ± 0.28, *P* = 0.002 (Unchanged)
19	*cj0355c*	*cosR*	Signal transduction, two-component	Upregulated	R = 1.69, *P* = 0.01 (Upregulated)	R = 1.65 ± 0.15, *P* = 0.01 (Upregulated)
20	*cj0551*	*Efp*	Cell envelope	Unchanged	R = 1.11, *P* = 0.35 (*P* value not acceptable)	R = 1.01 ± 0.15, *P* = 0.02 (Unchanged)

a No of spot in the 2D-electrophoresis gel (Fig. 3).

b Difference in expression from control to adapted CI 120.

c R: Difference in expression (R = expression of adapted cells/expression of control cells), *P*: parametric *P*-value.

d R: Difference in expression (R = expression of adapted cells/expression of control cells) expressed as the average ± standard deviation (*n* = 9), *P*: two-tailed *P* value.

e Could not be determined as the entire spot was treated as one for the purposes of quantification.

null: refers to genes that are putative and no name has been assigned to them.

## Discussion

The ability of bacteria to grow and survive in acidic environments is dependent on the regulation of the internal pH close to neutrality. Despite its importance in pathogenesis, the acid stress response in *C. jejuni* has not been studied to the extent that it has been for other food-borne pathogens. Little is known about how *C. jejuni* survives transit through the stomach, where it is exposed to low pH. However, the low number of *C. jejuni* cells required to initiate disease suggests that it can respond to acid shock. It has been shown that an ATR in *C. jejuni*, that is induced by acid and aerobic conditions, permits increased survival at lethal pH values (Murphy *et al*., 2003b), similar to *Salmonella typhimurium* (Foster, 1991), *Escherichia coli* (Raja *et al*., 1991) and *Aeromonas hydrophila* (Karem *et al*., 1994). In the present study, the evaluation of the induction of an acid + aerobic ATR of seven (7) *C. jejuni* strains prompted the further investigation of the ATR in the natural isolate CI 120 as it displays two very unique properties. First, under the conditions used, only CI 120 induced an ATR to acid + aerobic conditions in early stationary phase (Fig. 2A and B). The induction of the ATR in early stationary phase cells of *C. jejuni* is important because *Campylobacter* does not contain the genes that encode RpoS, the global stationary phase stress response mechanism (Kelly *et al*., 2001). Second, CI 120 has the unique property to confer acid tolerance to other *Campylobacter* strains through an extracellular component that provides a protective effect and is active from mid-exponential to early stationary phase (Murphy *et al*., 2003a). The reason why other strains lack the unique abilities of CI 120 is still unknown. A genetic marker (Single-nucleotide polymorphism [SNP] along with duplications, insertions and other polymorphisms) would have been useful and could be the purpose of a future study, together with the screening of a wide set of strains. In the present study, the induction of the ATR in CI 120 was further examined by microarray and proteomic approaches, comparing the gene expression profiles of adapted and control cells. Such gene expression profiles provide information on which genes and/or systems are expressed on adaptation to the acidic pH.

### ATR activates a number of stress responses

Acid stress in bacteria is known to induce cross-resistance to other stresses (e.g. heat shock, oxidative and osmotic stress), often by the induction of genes encoding proteins required to cope with these stresses (Bearson *et al*., 1997; Reid *et al*., 2008a). The microarray analysis showed that many genes involved in response to stress were upregulated. Among them, *cj0954c* (coding a putative DnaJ-like protein), *cj1107* (*clpS*) and *cj1108* (*clpA*). The stress response protein ClpA is a member of a family of molecular chaperones called the Clp ATPases (HSP100 proteins) which promote the ATP-dependent degradation of proteins (Schirmer *et al*., 1996). One very interesting result is that no difference was detected in the expression of the *cj0759* coding the universal chaperone DnaK (Table 2). This protein is known to be involved in the heat shock response, it is among the most highly conserved protein-coding genes known (Winter and Jakob, 2004) and has been identified as being induced in *S. typhimurium* under acid conditions (Foster, 1991). The same role has been attributed for GroEL, and yet, in the case of *C. jejuni* CI 120, it seems that there is no significant difference in the transcript levels of GroEL (*cj1221*) under the conditions used (Table 2). The results of this study are in contrast with a previous study where upregulation of *dnaK*, *groEL*, *grpE* and *clpB* was observed (Reid *et al*., 2008b). On the other hand, the same results are in accordance with another study (Birk *et al*., 2012) where no induction of heat shock proteins were observed. This might be due to the difference of the pH of the acid stress used in each study. In the first study (Reid *et al*., 2008b), a pH of 4.5 was used, whereas in the second study (Birk *et al*., 2012), a pH of 5.2 (similar to the adaptation pH in the current study) was used. This fact indicates the complexity of the different mechanisms required to protect cells against a lethal (4.5) or a sub-lethal (5.2) acid shock. It also indicates the difficulty in comparing the results from different studies, as they may not be consistent between them and might be strain and culture/media condition dependent, as indicated in Murphy *et al*. (2005). Previous studies in *C. jejuni* pointed out that GroEL and DnaK were not induced by starvation stress either (Klancnik *et al*., 2006).

Since the adaptation of *C. jejuni* CI 120 in pH 5.5 was performed under aerobic conditions, it is logical that factors playing a role during oxidative stress should be induced. The upregulation of numerous genes such as *cj0637c* (*mrsA*) and *cj1664*-*cj1665* (thioredoxins related with oxidative stress defense) (Holmes *et al*., 2005) shown by the transcriptome analysis, is in accordance with such induction. The MrsA protein has an important function as a repair enzyme for proteins that have been inactivated by oxidation (Boschi-Muller *et al*., 2000).

In *C. jejuni*, resistance to hydrogen peroxide is mainly mediated by the sole cytoplasmic catalase KatA, breaking it down to water and oxygen (Grant and Park, 1995). The iron-binding Dps protein (Cj1534c) also confers hydrogen peroxide stress resistance to *C. jejuni* (Ishikawa and Mizunoe, 2003). It protects DNA from oxidative damage by sequestering intracellular Fe^2+^ ions and storing them in the form of Fe^3+^ oxyhydroxide mineral. The connection between the iron-response and hydrogen peroxide/oxidative stress has been reported (Touati, 2000; Birk *et al*., 2012) and is reflected in the microarray results, which showed upregulation of numerous iron-related genes including *cj0334 (ahpC), cj1385* (*katA*), *cj1534c* (*dps*), *cj0175c* (*cfbpA*), *cj0174c* (*cfbpB*), *cj1659* (*p19*) and its putative ABC-transporter *cj1660-cj1663*. The fact that the most upregulated gene in this study was *cj1659* (*p19*), followed by the other iron transport-related genes, shows that iron plays an important role in *C. jejuni* gene regulation. The importance of iron in *C. jejuni* regulation is also indicated by the fact that *C. jejuni* has developed two similar and possibly overlapping regulatory systems which both use iron as the environmental signal; the ferric uptake regulator (Fur, connected with iron metabolism) and the peroxide stress regulator (PerR, connected with oxidative stress) (van Vliet *et al*., 2002).

### The role of the surface components in the ATR

*Campylobacter jejuni* surface structures affect virulence in several ways. Flagella are important for *C. jejuni* invasion of host cells by motility and by secretion of proteins (Larson *et al*., 2008). The role of flagella in the bacterial acid response remains unclear, and some gene expression studies have reported the upregulation of flagellar genes under conditions of acid stress (Merrell *et al*., 2001; Maurer *et al*., 2005) while others have reported the downregulation of these genes (Tucker *et al*., 2002; Wen *et al*., 2003). In the current study, genes related to motility that were differentially expressed (upregulated) were *cj0319* (*fliG*), *cj0320* (*fliH)* and *cj1334* (*maf3*). For the rest of the genes composing the flagella machinery, the results given by the microarrays are of no statistical value (*P*-value > 0.05).

Two-component regulators are part of the signal transduction system and are assumed to respond to changing host environments by regulating sets of genes that enhance the chances of bacterial survival. Two-component signal transduction systems (TCS) are comprised of a sensory histidine kinase (HK) which is located in the cytoplasm and a response regulator (RR) located in the cytoplasmic membrane (Brás *et al*., 1999). The sensor proteins of the TCS are assumed to provide external sensing, while the function of the RR is as an effector within the intricate regulatory network of the bacteria. In the genome of *C. jejuni* NCTC 11168 there are only five putative TCSs with an adjacent HK and RR (Parkhill *et al*., 2000). Several studies (Brás *et al*., 1999; MacKichan *et al*., 2004; Wösten *et al*., 2004; Raphael *et al*., 2005; Svensson *et al*., 2009) have shown that the TCSs are involved in colonization, while it is generally accepted that TCSs are significant in regulation of gene expression responses to environmental conditions. Only three out of the 12 *C. jejuni* two-component related genes (*cj1227c, cj1226c* and *cj0355c*; Table S1) were upregulated after an ATR response in CI 120. Cj0355c in particular, (CosR, Campylobacter oxidative stress regulation) is an OmpR-type oxidation stress response regulator, essential for *C. jejuni* viability (Garénaux *et al*., 2008). It regulates the expression of several oxidative stress proteins, including Dps, KatA and AhpC (Hwang *et al*., 2011) and the CmeABC multi-drug efflux pump (Hwang *et al*., 2012), showing once more the global response that the ATR has in *C. jejuni*. The role of *cj1227c* and *cj1226c* is still unknown. For the rest of the two-component related genes, the *P*-value from the microarrays was > 0.05, except for *cj0643* (CbrR, Campylobacter bile resistance regulator) whose expression remained unchanged (R = 0.77, *P* = 0.03). It is noteworthy that both *cj0355c* (*cosR*) and *cj0334* (*ahpC*) were upregulated in both protein and gene level (2D electrophoresis and microarrays results) in the current study.

*Campylobacter jejuni* is the first bacterium in which an *N-*linked protein modification system has been described (Weerapana and Imperiali, 2006; Young *et al*., 2007). The *N-*linked glycosylation pathway is responsible for post-translational modification of multiple proteins, and it has been proposed that *N-*linked glycosylation in *C. jejuni* is an important colonization determinant and might offer *C. jejuni* a system of immune invasion (Szymanski *et al*., 1999). In the present study, all the genes that comprise the protein glycosylation locus (*pgl*) were upregulated. This locus has been proposed as the *N*-glycan pathway, which has been suggested as being involved in chicken colonization (Kelly *et al*., 2006).

Adaptation to growth in a medium at low pH might be expected to involve the upregulation of genes encoding products capable of H^+^ extrusion. Consistent with this, ATR in *C. jejuni* CI 120 caused the upregulation of a putative Na^+^/H^+^ antiporter (Cj0832c). The Na^+^/H^+^ antiporters are integral membrane proteins that catalyse the exchange of H^+^ for Na^+^ in a manner that is highly dependent on the pH. In particular, the *E. coli* NhaA Na^+^/H^+^ antiporter (NhaA) protein probably functions in the regulation of the internal pH when the external pH is alkaline. It also uses the H^+^ gradient to expel Na^+^ from the cell and its activity is highly pH dependent (Padan *et al*., 2004).

### Energy metabolism

The remainder of the upregulated proteins were involved in metabolism. The upregulation of purine, pyrimidine and nucleotide protein synthesis indicates the increasing demand for energy and the synthesis of RNA and DNA. To meet all of its energy demands, *C. jejuni* utilizes oxidative phosphorylation. In the current study, all the genes of the transcriptional regulation of the *nuoA-N* gene locus encoding the proton-translocating reduced nicotinamide adenine dinucleotide (NADH):quinone dehydrogenase (complex I) were upregulated (Table S1). In *E. coli*, the subunits NuoL and NuoM are homologous to antiporters and have been implicated in proton pumping (Holt *et al*., 2003).The mutation of a number of NADH dehydrogenase complex (*nuoGIJN*) in *C. jejuni* led to impaired growth at low pH (Reid *et al*., 2008a).

### Final conclusions

**Figure 6:**
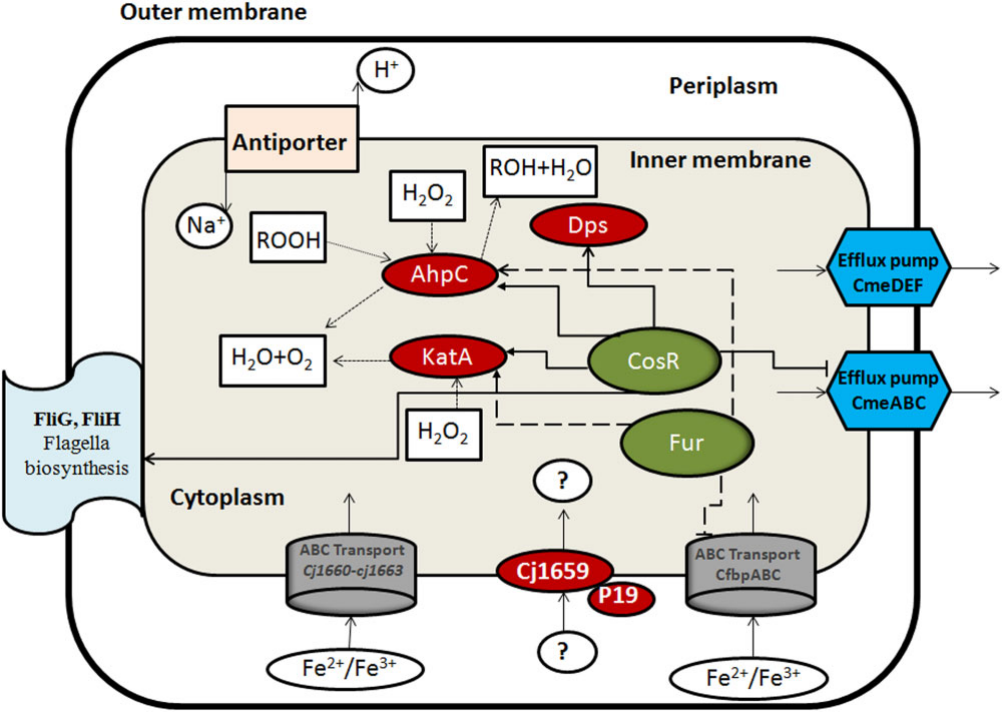
Overview of selected processes related with the ATR in *C. jejuni* under acid + aerobic treatment. ABC-transporter systems are symbolized by big grey barrels, efflux pumps by blue hexagons, single proteins by red ovals, regulators by green ovals, single transporter (anti-porter) by pink rectangular. Full list of genes is available in Supporting Information (Table S1).

In their natural environment, whether in the gastrointestinal tracts of avian or mammalian hosts or in the external environment during transmission, it is likely that *C. jejuni* cells are faced with growth-limiting or potentially lethal conditions, such as acidic and oxidative stresses. There is currently little information indicating how *C. jejuni* overcomes these stresses, either during the disease process through expression of virulence factor or by protective response to stress during transmission from one host to another. The results of this study demonstrate the ability of *C. jejuni* CI 120 to adapt to sublethal pH treatments in a manner that affects its survival and its ability to withstand subsequent stresses. This adaptation appears to involve a complex global response in a variety of biological pathways, inducing many different mechanisms, from energy metabolism to cell surface structures to iron acquisition and to oxidative stress. An overview of selected processes and related genes responsible for the global response of *C. jejuni* after an ATR is given in Fig. 6. The present work constitutes a first insight into the susceptibility on *Campylobacter* spp. to acid + aerobic treatments and the mechanisms leading to effective tolerance.

## Experimental procedures

### Terminology

In this study, four types of *C. jejuni* cultures have been used. Control cells: cultures grown microaerobically at 42°C to the appropriate growth phase; adapted cells: cultures grown microaerobically at 42°C to the appropriate growth phase, then the pH was reduced to 5.5 and the cultures were incubated at 42°C for 5 h; challenged cells: adapted cells challenged at pH 4.5; unadapted cells: cultures grown microaerobically at 42°C to the appropriate growth phase, then the pH was reduced directly at 4.5.

### Microorganisms and growth conditions

Seven strains of *C. jejuni* were used during this study. Strains CI 120, CI 151 and CI 195 were isolated from poultry during processing, NCTC 11351 and NCTC 81116 were obtained from the National Collection of Typed Cultures (Central Public Health Laboratory, London, UK), CN 26 was isolated from a wild bird in Finland and CN 107 was an ovine isolate. All natural isolates were identified by API Campy (bioMerieux) and by a PCR/DNA probe membrane-based colorimetric assay (O'Sullivan *et al*., 2000). The three poultry isolates were in the culture collection at the Department of Microbiology NUI, Galway. *Campylobacter jejuni* CN 26 and CN 107 (Campynet collection strains) were obtained from Queen's University, Belfast. All strains were maintained at −20°C in Tryptone Soy Broth supplemented with 20% glycerol. Strains were routinely grown at 42°C in a multi-gas incubator under microaerobic conditions (5% O_2_, 10% CO_2_ and 85% N_2_).

### Assay of acid tolerance and induction of an ATR

The induction of an ATR (5 h at pH 5.5 under aerobic conditions) and the measurement of acid tolerance were undertaken according to Murphy *et al*. (2003b). For all *C. jejuni* strains used, cultures were grown microaerobically to the appropriate growth phase in Brucella Broth at pH 7.0 (control cells). The pH of the culture was reduced to 5.5 by direct addition of 0.5 M HCl and left at 42°C for 5 h in aerobic conditions (adapted cells). Cells were subsequently challenged at pH 4.5 (challenged cells) and serial dilutions were performed in maximum recovery diluent (MRD, Oxoid). Survivors were calculated by plating the cell suspensions on Campylobacter Blood Free Selective Agar plates (CCDA). Plates were incubated for 48 h at 42°C under microaerobic conditions. Colonies were counted and the numbers of colony forming units per ml were determined.

### Preparation of the cultures for proteomics and microarrays

*Campylobacter jejuni* cultures were grown in Brucella Broth at pH 7.0. According to growth curves, after 18 h at 42°C under microaerobic conditions, cultures had achieved early stationary phase. Half of the culture was collected by centrifugation (10 000x *g*/4°C/15 min; control cells). To the other half of the culture, the pH was decreased to 5.5, incubated aerobically at 42°C for 5 h and then harvested by centrifugation (10 000x *g*/4°C/15 min; adapted cells).

### Cytosolic protein cell lysates for 2D electrophoresis

The *C. jejuni* cells used for protein extraction were adapted under the conditions described above. Adapted and control cells (1 l each) were harvested (10 000x *g*/4°C/15 min), washed twice with 40 mM Tris-base pH 9.5 and re-suspended in 10 ml of chilled extraction solution [8 M urea, 2 M thiourea, 2% 3-(3-cholamidopropyl-dimethylammino)-1-propane-sulfonate (CHAPS), 2% 3-(decyldimethyl-amino)-propanesulfonate (SB 3–10), 1% Triton X-100, 150 U Endonuclease, 1 mM phenylmethylsulfonyl fluoride (PMSF) and 20 mM dichlorodiphenyltrichloroethane (DTT)]. PMSF and DTT were added to the extraction solution just prior to use. Cells were lysed using 0.1 mm silica/zirconium beads in a mini-bead beater for 3 min in three cycles of 1 min followed by 2 min cooling in iced water. Insoluble proteins were precipitated by centrifugation (45 000x *g*/4°C/30 min). The supernatant was recovered and the concentration of the soluble proteins was determined by a commercial copper-binding protein assay (PlusOne 2-D Quantkit, GE Healthcare) using BSA as the standard. The absorbance was measured at 480 nm. Samples were stored at −20°C until analysed.

### Sample preparation for 2D electrophoresis

Proteins (250 μg/ml) were precipitated from the cell lysates with four volumes of ice-cold acetone for 2 h at −20°C, centrifuged (10 000x *g*/4°C/10 min) and air dried. The precipitated proteins were solubilized with 350 μl of rehydration solution (Genomic Solutions) supplemented with 8 μl 2% isopropyl β-D-1-thiogalactopyranoside (IPG) buffer (GE Healthcare) and 2.5 mg/ml DTT. Samples were incubated at room temperature for 2 h in a sample mixer (Dynal Biotech). To ensure complete solubilization, samples were sonicated in two cycles of 10 s followed by 2 min cooling in iced water.

### 2D electrophoresis

Two-dimensional electrophoresis of soluble proteins was performed using an HT Investigator apparatus (Genomic Solutions) according to the manufacturer's instructions. Samples were applied to 18 cm IPG strips (pH range 3–10 or 4–7; GE Healthcare) in a re-swelling tray (Genomic Solutions). Following rehydration, samples were focused at 20°C with an initial voltage of 500 V for 250 Vh (30 min) and then focused for 90 kVh (18 h), with a gradual voltage increase to 5000 V. After focusing, strips were equilibrated for 10 min in equilibration buffer (45 mM Tris-HCl pH 7.0, 6 M urea, 30% glycerol, 2% SDS and 0.01% Bromophenol blue) containing 10 mg/ml DTT, followed by 10 min in equilibration buffer containing 25 mg/ml iodoacetamide. Strips were placed on top of the second dimension gel (precast Tris-Tricine/10% Duracryl gels, Genomic Solutions), and electrophoresis was run at 500 V and 14 000 mW per gel for 5 h with Tris/Acetate (40 mM Tris-HCl, 20 mM acetic acid, 1 mM EDTA, pH 8.0; lower buffer) and Tris/Tricine/SDS (10 m Tris-HCl, 10 mM Tricine, 0.01% SDS, pH 8.3; upper buffer). Wide molecular weight range markers (Sigma) were applied at the acidic end of the IPG strips. Gels were fixed and stained for 16 h with Coomassie Brilliant Blue-G (Sigma) and de-stained first with 10% acetic acid in 25% methanol, for 15–30 s and followed by 25% methanol for 10 to 16 h. The gel images were captured using a Kodak Image station 440 CF and the 2D maps of cytosolic proteins were analysed using HT InvestigatorSoftware (Version 2.1, Genomic Solutions). Gels in Fig. 3 were obtained by averaging the spot analysis of the gels from three independent adapted early stationary phase cultures and comparing it to the analysis of the gels obtained from three independent control early stationary phase cultures, using protein-spot number 11 (CheW) for normalization (Fig. 3). Using the image analysis software HT Investigator (Version 2.1, Genomic Solutions), only proteins that showed reproducible changes in expression in all three independent experiments were highlighted on the averaged gel.

### Matrix assisted laser desorption ionization-time-of-flight mass spectrometry and peptide mass fingerprint analysis for protein identification

Protein spots were excised from the 2D-electrophoresis gels, introduced to a Genomic Solutions ProGest semiautomatic enzyme digestor where they were washed, in-gel reduced, S-alkylated and in-gel digested with trypsin. After digesting for 8 h, the peptide extract produced was dried in Savant speed-vac and re-dissolved in 10% formic acid and transferred to a Genomic Solutions ProMS. The peptide extract produced by the in-gel cleavage was passed through a Zip Tip (ZTC18096 Millipore). The adsorbed peptides were washed three times with 0.01% formic acid and then eluted in 2 μl of a 1% solution of α-cyano-4-hydroxycinnamic acid in 70% acetonitrile/30% formic acid on to a mass spectrometer sample plate. The mass spectra were acquired on an Applied Biosystems Voyager-DE STR matrix-assisted laser desorption ionization-time-of-flight mass spectrometry (MALDI-TOF-MS). The instrument was operated in the reflectron-delayed extraction mode as per manufacturer's recommendations. Spectra were internally calibrated using trypsin auto-digestion products. A non-redundant protein sequence database (Matrix Science Mascot) was used for database searches using the peptide search software package. The search parameters used were as follows: Cysteine as S-carbamidomethyl-derivative; maximum allowed peptide mass error between 50 ppm and 150 ppm; more than five peptide mass hits required for a protein match. No restriction was placed on either the isoelectric point or species of origin of the protein. A protein mass range between 0 and 150 kDa was allowed.

### RNA extraction and preparation for microarrays

Total RNA was isolated from adapted and control *C. jejuni* CI 120 cells (1 l each) using the Qiagen RNeasy Maxi Kit (Cat No 75162), following the manufacturer's protocol. The yield of total RNA was typically about 100 μg from 1 l of culture. Total RNA was quantified using a NanoDrop-1000 spectrophotometer, and its integrity was analysed using an Agilent 2100 Bioanalyser. Total RNA was considered suitable for this study if the 23S/16S rRNA was > 1.2. Ten (10) μg of total RNA was converted to enrich messenger RNA (mRNA) using the Applied Biosystems (Ambion) MicrobExpressTm Kit (Cat No AM1905). The MicrobExpress Extension module was used and the manufacturer's protocol was followed. The expected yield of enriched mRNA from 10 μg of total RNA was typically 1.0–2.5 μg. Enriched mRNA (200 ng) was converted to cRNA using the Applied Biosystems (Ambion) MessageAmp II-Bacteria RNA Amplification Kit (AM1790). Amino-allyl-UTP was incorporated into the cRNA during the *in vitro* transcription (IVT) reaction. The manufacturer's protocol was followed. The expected yield from 200 ng was about 100 μg. For the fluorescent labeling, Alexa Fluor 555 (Invitrogen A32756) was coupled to cRNA following the manufacturer's instructions. Unincorporated dye was removed using RNeasy Mini Columns (Qiagen 74104). The Qiagen quick clean-up protocol was followed. Fluor labeled cRNA was then fragmented to a mean size of 150 nt (MYcroarray proprietary protocol). The mean fragment size was verified with an Agilent 2100 Bioanalyser.

### cRNA microarrays

The microarray slides were manufactured by MYcroarray (5692 Plymouth Road, Ann Arbor, MI 48105, USA). Each slide contained three arrays and each array contained three identical replicates of each *C. jejuni* NCTC 11168 probe sequence. Three biological replicates (RNA extracted from three independent cultures of adapted and control *C. jejuni* CI 120) were used. Hybridization and washing of the slides was performed following the manufacturer's protocol. The arrays were scanned in an Axon 4000B Scanner (Molecular Devices) set at 5 μm per pixel resolution. The photomultiplier tube (PMT) setting was adjusted to appreciate the maximum dynamic range of signal (0 to 65 000). This was accomplished by adjusting the PMT until just a few spots had some saturated pixels.

### Microarray data analysis

Scanned images were quantified using GenePixProSoftware (version 6.1.0.4). For signal extraction, circular feature indicators (30 μm diameter) were centered over each spot. Median feature pixel intensity was extracted. A global background value, specific for each array, was subtracted from the median pixel signal value for each *C. jejuni* spot. The global background value was arbitrarily set to the fifth percentile darkest *C. jejuni* spot. Local background was not used due to the array manufacturer's process. After background subtraction, a signal value of zero was substituted for negative values. Finally, the entire background corrected C. *jejuni* signal distribution was shifted to the right by adding a constant value of 10. To adjust for differences in chip to chip brightness, a scale factor was created to equalize signal across all arrays. Only features that met the following criteria were used to calculate the scale factor: *C. jejuni* spots must have less than 10% saturated pixels; the median signal had to be more than four-fold background; the normalization spot had to qualify in all six arrays. There were 9166 qualifying spots. The average signal for these qualifying spots, called normalization features (NF), was calculated for each array. A scale factor (SF) for each array was calculated using the following equation (1):

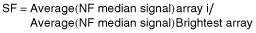
(1)

For each array, the background corrected median pixel signal for all *C. jejuni* probe sequences was then adjusted by multiplying by the SF. Replicate probe SF adjusted median signal was then averaged to yield a final adjusted signal value for each unique probe sequence (gene). To identify genes that may be differentially expressed between the control and adapted experimental conditions, the Student's *t*-test *P-*value and fold change ratio were used in combination. Two arbitrary differential expression thresholds were used; Student's *t*-test *P* < 0.05 and a fold change ratio of > 2 (or < 0.5; adapted/control) or Student's *t*-test *P* < 0.01 and a fold change ration of > 1.5 (or < 0.66).

### QRT-PCR

Total RNA was isolated from 100 ml *C. jejuni* CI 120 adapted and control cultures, using the PureYield RNA Midiprep System (Promega, Cat No Z3741) and following the manufacturer's protocol. The yield of total RNA was typically about 10 μg out of 100 ml of culture. Complementary DNA (cDNA) was synthesized from 1 μg of total RNA, using the QuantiTech Reverse Transcription Kit (Quiagen, Cat No 205311), which involves an integrated removal of Genomic Deoxyribonucleic Acid (gDNA) contamination. Synthesized cDNA was marked with SYBR Green using the QuantiTech SYBR Green PCR Kit (Quiagen, Cat No 204141) adding 2.5 ng/μl of cDNA and 500 nM of each primer. In all cases, the manufacturer's protocol was followed. The specificity of each primer pair was determined by conventional PCR. The sequences of the primers are given in Supporting Information (Table S3). Total RNA and cDNA were quantified with the use of a NanoDrop-1000. QRT-PCRs were performed using a LightCycler 480 (Roche). The reaction conditions were: 3 min at 95°C, followed by 40 cycles of 10 s at 95°C, 20 s at 58°C and 30 s at 72°C. Results are the means of three independent experiments, all performed in triplicate i.e. *n* = 9 for each one. To identify genes that may be differentially expressed between the control and adapted experimental conditions, the same criteria as for microarrays were applied. The efficiency (E) of the QRT-PCR reactions were calculated as described in the instrument operator's manual. In summary, standard curves were plotted and the efficiency was calculated from the given slopes, according to the equation: E = 10^−1/slope^.

### Microarray data accession numbers

Microarray data are available in the ArrayExpress database (www.ebi.ac.uk/arrayexpress) under accession number E-MEXP-3835.

## Supporting Information

**Table S1.** Genes differentially expressed (upregulated) in *C. jejuni* CI 120 using microarrays. The complete list.

**Table S2.** Genes differentially expressed (downregulated) in *C. jejuni* CI 120 using microarrays. The complete list.

**Table S3.** Oligonucleotides used as primers for QRT-PCR assays.
