# Supplementary material for: The acid adaptive tolerance response in Campylobacter jejuni induces a global response, as suggested by proteomics and microarrays
Source: Microb Biotechnol. 2015 Jul 29;8(6):974–88. doi: 10.1111/1751-7915.12302 (PMC4621450; doi:10.1111/1751-7915.12302)
Supplement: Supplementary file 2 — Table S2. Genes differentially expressed (downregulated) in C. jejuni CI 120 using microarrays. The complete list. [file mbt20008-0974-sd2.docx]

**Table S2:** Genes differentially expressed (down-regulated) in *C. jejuni* CI 120 using microarrays. The complete list.

| **Gene locus** | ***Gene name*** | **Description** | **Pathway/Class/Function** | **R^(a)^** | **Parametric *P*-value** |
| --- | --- | --- | --- | --- | --- |
| *cj0103* | *atpF* | ATP synthase | Oxidative phosphorylation | 0.65 | 0.01 |
| *cj0200c* | null | Putative periplasmic protein | Unknown | 0.40 | 0.009 |
| *cj0494* | null | Putative exporting protein | Unknown | 0.49 | 0.02 |
| *cj0606* | null | Putative secretion protein | Efflux pump | 0.45 | 0.01 |
| *cj1004* | null | putative periplasmic protein | Unknown | 0.42 | 0.04 |
| *cj1190c* | *cetA* | Bipartite energy taxis response | Chemotaxis | 0.53 | 0.01 |
| *cj1356c* | null | Putative integral membrane protein | Unknown | 0.47 | 0.04 |
| *cj1622* | *ribD* | Riboflavin-specific deaminase/reductase | Roboflavin metabolism | 0.43 | 0.04 |
| *cj1623* | null | Putative membrane protein | Unknown | 0.46 | 0.001 |
| *cj1625c* | *sdaC* | Amino acid transporter | Membrane transporter | 0.53 | 0.01 |
| *cj1668c* | null | Putative periplasmic protein | Unknown | 0.50 | 0.009 |

(a): Difference in expression (R=Expression of adapted cells/expression of control cells)
