# Supplementary material for: The acid adaptive tolerance response in Campylobacter jejuni induces a global response, as suggested by proteomics and microarrays
Source: Microb Biotechnol. 2015 Jul 29;8(6):974–88. doi: 10.1111/1751-7915.12302 (PMC4621450; doi:10.1111/1751-7915.12302)
Supplement: Supplementary file 3 — Table S3. Oligonucleotides used as primers for QRT-PCR assays. [file mbt20008-0974-sd3.docx]

**Table 1.** Oligonucleotides used as primers for QRT-PCR assays

| **Gene** | **Primer** | **Sequence (5' -› 3')** |
| --- | --- | --- |
| *cj0193c* | #Cj0193c-F | GTTTTGAAGATGGTATGGTGGGAATG |
|  | #Cj0193c-R | AATACTGCATCTTTACCCGCTAAATGC |
| *cj0533* | #Cj0533-F | CATGGACTTGAAGTAGCGAGAGTTTTG |
|  | #Cj0533-R | AGGGCATAAAAATCCCCTTCTGC |
| *cj1317* | #Cj1317-F | CAACGGGCATTGCTACAGAAGAAG |
|  | #Cj1317-R | AAAGCTATGATCACTTAGTCCCACTTCAAC |
| *cj0334* | #Cj0334-F | CGGTATTGAAGTAATTGGAATTTCAGGAG |
|  | #Cj0334-R | CAAGAACGAACCACGAAGGGC |
| *cj0355c* | #Cj0355c-F | CAAACTGGACTTTACCTGATGGAGATG |
|  | #Cj0355c-R | CTTGCTTCAATTCTTGCGAGTAGAATATC |
| *cj0551* | #Cj0551-F | TATGGATACGCAAACATATGAACAAGTAGC |
|  | #Cj0551-R | GCTTTACCATTATGGAAAAGCACATCTAC |
| *cj1595* | #Cj1595-F | GGCTATGTACCAAGCGAAGAAATTAAAG |
|  | #Cj1595-R | GGAGTGATTTGTCCATCAGTTGTTACG |
| *cj0759* | # Cj0759-F | GATCTAGGTGGGGGAACTTTTGACG |
|  | # Cj0759-R | CTGCTTCTTTCAAGCGTTGCAAAG |
| *cj0538* | # Cj0538-R | GGGAAGTAGATTTTATGCTTTCGACTGC |
|  | # Cj0538-F | CCACATTTCCTACCTCATCTTTAGCTATG |
| *cj1221* | # Cj1221-R | GCTGAAGATATTGAAGGTGAAGCGC |
|  | # Cj1221-F | CAAGTTCTTCAGAAATCACTTCTCCACC |
| *cj0559* | # Cj0559-R | GAGCACAATATAGAAGTTGAATTTGGTTCTG |
|  | # Cj0559-F | TTTGTTAAAGTCATAGGAAGTTTGTAATCAGG |
| *cj0806* | #Cj0806-R | GGATAAAAATATTATCATTGGGGCGATGACGGC |
|  | #Cj0806-F | CGATTTATGTATCGGCAAAATTATCCTTATTCCTAATG |
| *cjr01* | #Cjr01-F | GAAAGCGTGGGGAGCAAACAG |
|  | #Cjr01-R | GTTTTAATCTTGCGACCGTACTCCC |

^a^: served as endogenous control (housekeeping genes)
